# Performing whole-exome sequencing in Bardet-Bield syndrome

**DOI:** 10.1186/2046-2530-4-S1-P11

**Published:** 2015-07-13

**Authors:** S Castro-Sánchez, M Alvarez-Satta, D Valverde

**Affiliations:** 1 Biochemistry, Genetics and Immunology, University of Vigo, Vigo, Spain; 2 Instituto de Investigación Biomédica Ourense-Pontevedra-Vigo (IBI), Vigo, Spain

## Objective

Bardet-Biedl syndrome (BBS) is a rare disease characterized by a high genetic heterogeneity, accounting for 75% of affected families. As part of the next-generation technology, whole-exome sequencing (WES) allows all exons of the genome to be sequenced at once. Here we show the use of WES as a useful approach in BBS families in which mutations in predominant genes have been discarded.

## Methods

We studied the exome of 15 unrelated patients clinically diagnosed with BBS. For this purpose, we used the Nimblegen SeqCap v3 (64Mb) kit for exome capture, followed by the use of Illumina HiSeq 2000 sequencer, with a mean coverage per sample >50X. Once the sequencing data alignment and variant calling were made, we carried out a filtering strategy to identify candidate mutations responsible for the disorder, which need to be confirmed by direct sequencing. Segregation analyses were performed when possible.

## Results

This approach, focused on evaluating mutations in genes involved in the pathology (all *BBS *genes, and also *ALMS1 *gene), has allowed us to diagnose 4 families, identifying 5 potential disease-causing mutations representing 3 different known genes (*BBS2, BBS5 *and *ALMS1*). 4 of these changes were novel mutations and segregation studies confirmed the carrier state in parents. In the remaining families, we have selected some candidate genes which are under evaluation.

## Conclusion

We consider this is a good and economically worthwhile strategy when predominant mutations have been discarded in the analysed population. Moreover, it allows us to reduce diagnostic time and offers the possibility to identify novel candidate genes.

